# Navigating the Enigma: A Comprehensive Review of Idiopathic Intracranial Hypertension

**DOI:** 10.7759/cureus.56256

**Published:** 2024-03-16

**Authors:** Saket S Toshniwal, Jiwan Kinkar, Yatika Chadha, Kashish Khurana, Harshitha Reddy, Abhinav Kadam, Sourya Acharya

**Affiliations:** 1 Medicine, Jawaharlal Nehru Medical College, Datta Meghe Institute of Higher Education & Research, Wardha, IND; 2 Neurology, Jawaharlal Nehru Medical College, Datta Meghe Institute of Higher Education & Research, Nagpur, IND; 3 Psychiatry, Jawaharlal Nehru Medical College, Datta Meghe Institute of Higher Education & Research, Wardha, IND

**Keywords:** complications, management strategies, visual disturbances, elevated intracranial pressure, pseudotumor cerebri, idiopathic intracranial hypertension

## Abstract

An unidentified source of increased intracranial pressure is a hallmark of idiopathic intracranial hypertension (IIH), also referred to as pseudotumor cerebri. It mainly affects young, obese women, yet it can happen to anyone, regardless of age, gender, or weight. IIH presents with symptoms such as headaches, visual disturbances, and pulsatile tinnitus and can lead to severe complications, including vision loss, if left untreated. Diagnosis involves clinical evaluation, neuroimaging, and lumbar puncture, while management options include medical interventions and surgical procedures. This review provides a comprehensive overview of IIH, including its etiology, clinical presentation, epidemiology, complications, management approaches, and challenges. Increased awareness among healthcare professionals, standardized diagnostic criteria, and further research efforts are essential for improving outcomes and quality of life for individuals with IIH.

## Introduction and background

Idiopathic intracranial hypertension (IIH), also known as pseudotumor cerebri, is a neurological disorder characterized by increased intracranial pressure (ICP) without an identifiable cause [1]. It primarily affects young obese women of childbearing age but can occur in individuals of any age, gender, or weight. The hallmark feature of IIH is elevated ICP, leading to symptoms such as headaches, visual disturbances, and pulsatile tinnitus. Despite its name, IIH can lead to significant morbidity and even blindness if not properly managed [2].

IIH poses a significant challenge to both patients and healthcare providers due to its unpredictable course and potential for severe complications. Early recognition and appropriate management are crucial to prevent vision loss and improve the quality of life (QOL) for affected individuals [3]. Understanding the underlying mechanisms of IIH and optimizing treatment strategies are essential for minimizing its impact on patients’ health outcomes [4].

This review aims to provide a comprehensive overview of IIH, including its etiology, clinical presentation, epidemiology, complications, management approaches, and current challenges. By synthesizing the existing literature and highlighting key findings, this review aims to enhance understanding of IIH among healthcare professionals, facilitate early diagnosis and intervention, and ultimately improve outcomes for patients with this challenging condition.

## Review

Etiology and pathophysiology

Primary Factors Contributing to IIH

**Figure 1:**
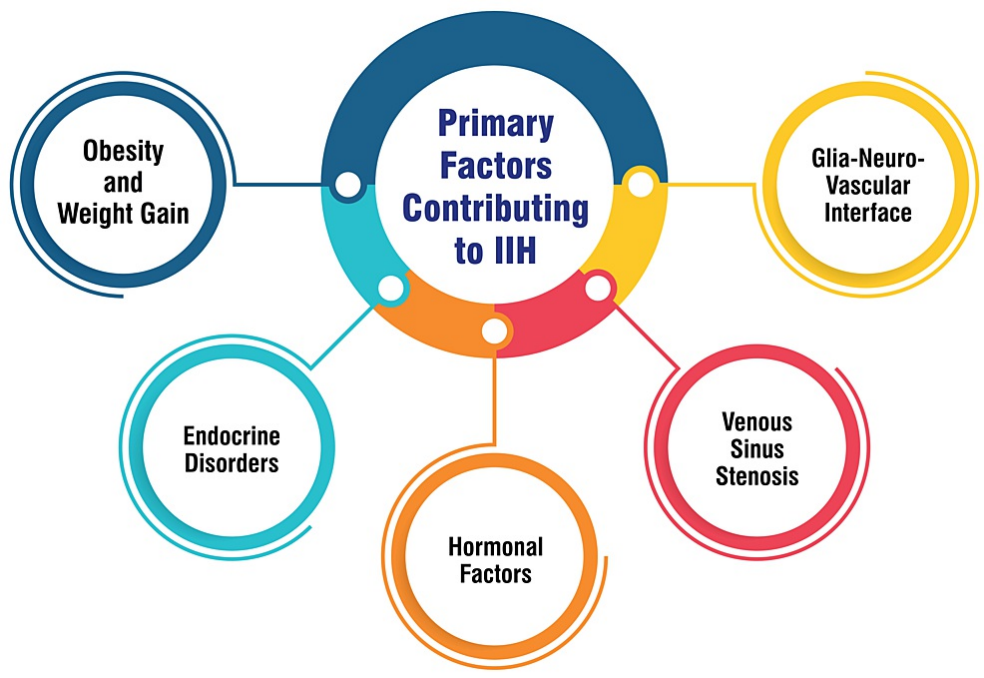
Primary factors contributing to IIH IIH, idiopathic intracranial hypertension Image credit: Saket S. Toshniwal

IIH is more prevalent among women of reproductive age. It is often correlated with obesity and weight gain, characterized by a higher BMI or recent weight gain [5]. Additionally, endocrine disorders such as Addison’s disease, hypoparathyroidism, and steroid withdrawal have been identified as potential contributors to IIH [6]. Hormonal fluctuations during pregnancy, menopause, or the use of hormonal contraceptives may also play a role in the development of IIH [5]. Moreover, intracranial venous hypertension resulting from venous sinus stenosis has been proposed as a primary factor underlying IIH [7]. Recent research has shed light on the involvement of the glia-neuro-vascular interface within the brain in the pathophysiology of IIH [8]. This interface involves complex interactions between glial cells, neurons, and capillaries, suggesting a novel avenue for understanding the mechanisms driving increased ICP in IIH. Other potential risk factors for IIH include genetic predisposition, metabolic disorders, and certain medications such as nalidixic acid, lithium, and systemic lupus erythematosus [6]. However, the associations of these factors with IIH are less firmly established than those of the primary factors mentioned above. Continued research is warranted to elucidate these risk factors’ role and further refine our understanding of the etiology and pathogenesis of IIH. The primary factors contributing to IIH are shown in Figure 1.

Mechanisms Underlying Increased ICP

Elevated ICP can arise from various factors, including space-occupying lesions, obstruction of CSF flow, and stenosis of the venous sinuses [9]. The correlation between ICP and intracranial volume is delineated by a sigmoidal pressure-volume curve, indicating that expansion of volume up to 30 cm³ typically elicits negligible alterations in ICP due to compensatory mechanisms such as venous blood extrusion from the cranium [9]. Once these compensatory mechanisms are depleted, further volume increases can precipitate substantial elevations in ICP [9].

In the context of IIH, the precise mechanisms underlying elevated ICP still need to be fully elucidated. However, it is theorized that dysregulation of CSF dynamics and metabolic and hormonal factors may contribute to the condition’s onset [10]. The interaction among these factors and their impact on the glia-neuro-vascular interface represent ongoing areas of investigation within the realm of IIH [7].

Elevated ICP may stem from diverse factors, including space-occupying lesions, CSF flow obstruction, and venous sinus stenosis. The relationship between ICP and intracranial volume is depicted by a sigmoidal pressure-volume curve, wherein volume expansion up to 30 cm³ typically results in negligible changes in ICP owing to compensatory mechanisms. The precise mechanisms underlying elevated ICP in IIH are not fully understood. Still, it is believed that dysregulation of CSF dynamics and metabolic and hormonal factors may contribute to the development of the condition [11].

Potential Genetic and Environmental Influences

IIH is a multifaceted disorder influenced by both genetic and environmental factors. The involvement of genetic elements in IIH development is indicated by familial occurrences of the condition and the identification of candidate regions on chromosomes 5, 13, and 14 in a genome-wide association study [12]. Nevertheless, a definitive Mendelian inheritance pattern has not been established, suggesting that multiple genes, each with a modest effect, likely contribute to IIH development [12]. Environmental factors, notably obesity and weight gain, also significantly influence IIH risk. Obesity, particularly prevalent among women of childbearing age, stands as a well-established risk factor for IIH [6]. The interplay between genetic and environmental factors in IIH etiology mirrors patterns observed in other neurological disorders [12]. Furthermore, familial cases of IIH underscore the familial aggregation of the condition, with some families reporting multiple affected members [13]. Yet, the precise genetic underpinnings of these familial cases remain incompletely understood. Continued research efforts are imperative to unravel the intricate interplay between genetic and environmental factors in IIH development and to pinpoint potential therapeutic targets for this complex condition.

Clinical presentation

Symptoms and Signs of IIH

**Figure 2:**
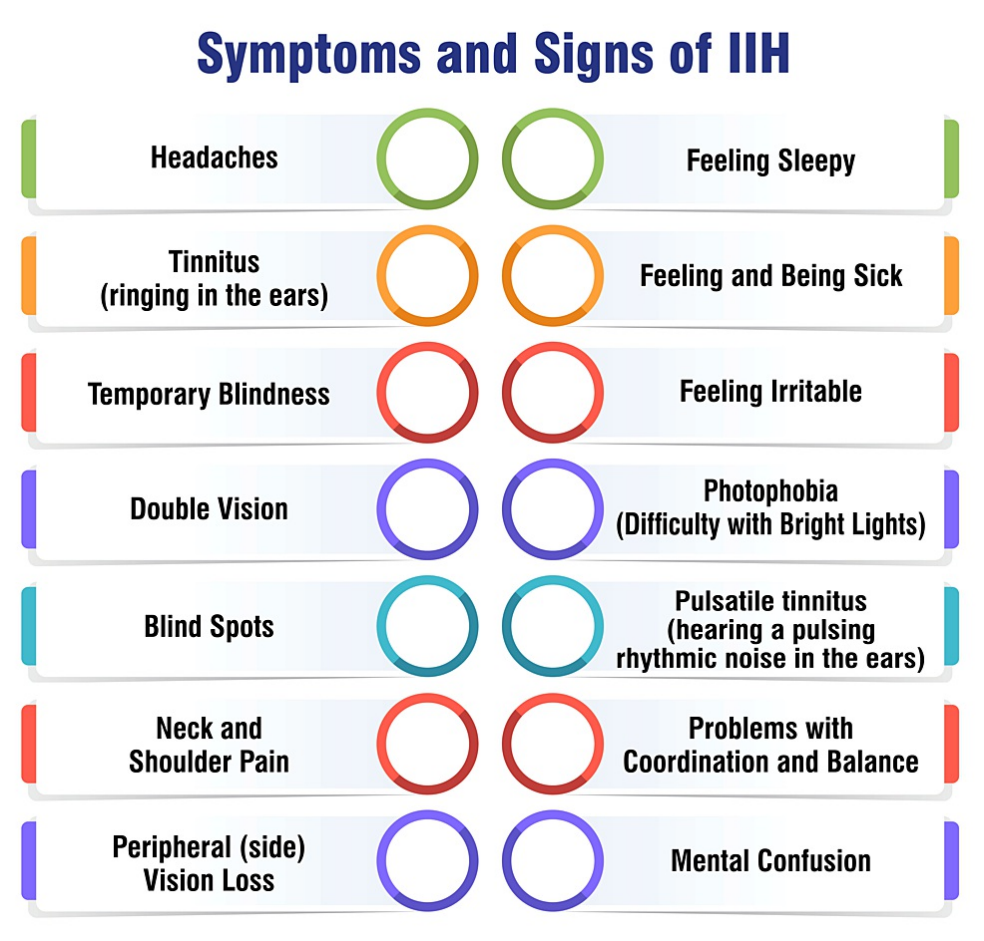
Symptoms and signs of IIH IIH, idiopathic intracranial hypertension Image credit: Saket S. Toshniwal

IIH is characterized by increased pressure around the brain, leading to various symptoms that can mimic those of a brain tumor. The symptoms of IIH are presented in Figure 2 [5].

Diagnostic Criteria and Methods

Clinical evaluation is paramount in the diagnosis of IIH, as patients typically present with symptoms related to increased ICP and papilledema. These symptoms commonly include headaches, transient visual obscurations, pulsatile tinnitus, a subjective decrease in vision, and horizontal diplopia [13]. Brain imaging plays a crucial role in the evaluation process, with venography being essential to exclude structural causes and venous sinus thrombosis. MRI or CT scans are also recommended to assess for any intracranial abnormalities [14].

Another critical aspect of the diagnostic workup is a lumbar puncture, which is crucial for confirming IIH. Lumbar puncture reveals an opening pressure greater than 250 mm CSF with normal constituents. Measuring CSF opening pressure is one of the essential requirements for establishing the diagnosis of IIH [14]. Papilledema evaluation is equally critical in diagnosing IIH and involves identifying papilledema while excluding intracranial causes of raised ICP through appropriate brain imaging. Fundus examination aids in assessing the degree of papilledema, which can vary among patients [15].

In addition to these diagnostic procedures, it is essential to consider the patient’s medical history and perform a detailed physical examination, including a visual acuity and color vision assessment. Visual field testing should also be conducted to evaluate the impact of IIH on visual function [15]. A comprehensive approach to clinical evaluation, brain imaging, lumbar puncture, and papilledema assessment is essential for accurately diagnosing IIH and initiating appropriate management strategies.

Differential Diagnosis Considerations

When considering the potential causes of IIH, clinicians must thoroughly investigate to rule out any external factors contributing to the elevated ICP. Exogenous substances or medications, as well as various systemic and neurological disorders, can manifest similarly to IIH. If the increased ICP is attributed to an underlying cause, patients are categorized under the broader diagnosis of pseudotumor cerebri. Conditions associated with pseudotumor cerebri encompass obstructive venous disorders, endocrine abnormalities, the intake of certain exogenous substances or medications, infectious and post-infectious states, and other medical conditions [16]. Taking a comprehensive medical and medication history focused on symptoms suggestive of obstructive sleep apnea, exogenous substance use, and underlying systemic or neurological disorders is crucial for achieving an accurate diagnosis specific to IIH [17]. Common symptoms indicative of elevated ICP in IIH patients typically include headaches, transient visual obscurations, pulse-synchronous tinnitus, subjective decrease in vision, horizontal diplopia, dizziness, photophobia, neck pain, and radicular pain [17]. Furthermore, papilledema, characterized by bilateral disc edema resulting from increased ICP, is a significant physical finding in IIH patients [17].

Epidemiology

Prevalence and Incidence Rates

The IIH epidemiology reveals intriguing patterns across various populations. In the United States, data from 1997 to 2016 indicate an estimated national annual incidence of IIH at 1.15 per 100,000 individuals, with a notable increase observed over the years [18]. This condition predominantly affects women, with an incidence rate of 1.97 compared to 0.36 in males [6]. The highest incidence is observed among individuals aged 18 to 44, with a rate of 2.47 per 100,000 individuals [6]. Socioeconomic disparities are evident, with low-income patients exhibiting a higher incidence compared to those with middle or high incomes [18].

When examining racial and ethnic groups within the United States, notable variations in IIH incidence emerge. Blacks have the highest incidence rate at 2.05, followed by Whites at 1.04, Hispanics at 0.67, and Asian/Pacific Islanders at 0.16 per 100,000 individuals [18]. These disparities underscore the significance of considering demographic factors when assessing the prevalence of IIH.

On a global scale, the prevalence of IIH is on the rise, largely attributable to the obesity epidemic, particularly impacting women of childbearing age [6]. Recognizing risk factors associated with IIH, such as obesity and certain endocrine disorders, is crucial for devising effective management and treatment strategies [6]. Furthermore, distinguishing IIH from other conditions that can mimic its symptoms is essential to ensure appropriate care and favorable outcomes for patients [6].

Demographic and Risk Factor Associations

Demographic and risk factor associations with IIH have been extensively researched. The condition exhibits a higher prevalence among females, with an incidence of 1.97 per 100,000 individuals among females compared to 0.36 per 100,000 among males [18]. IIH is more commonly observed in younger age groups, with the highest incidence noted among individuals aged 18 to 44 years [18]. Interestingly, low-income patients demonstrate a higher incidence of IIH compared to those with middle or high incomes [18]. Furthermore, IIH prevalence varies among racial and ethnic groups, with a higher incidence among Blacks, followed by Whites, Hispanics, and Asian/Pacific Islanders [18]. Notably, there is no discernible difference in IIH incidence between urban, suburban, or rural communities [18]. Obesity stands out as a significant risk factor for IIH, with an increased likelihood observed among low-income, female, young, and Black patients [18]. Additionally, IIH is associated with a two-fold increase in cardiovascular disease risk among women [19]. Other notable risk factors include endocrine disorders, steroid withdrawal, and certain medications [6]. Understanding these demographic and risk factor associations is essential for effective management and prevention strategies targeting high-risk populations.

Geographical Variations

IIH displays geographical variations in both its prevalence and incidence rates. Studies have revealed notable regional disparities in the female-to-male ratio among IIH patients [20]. Furthermore, IIH appears to be more prevalent among Black and Hispanic women, a trend that persists even when considering the demographics of metropolitan areas [21]. This association remains significant even after accounting for obesity, low income, and access to healthy food options [21]. In the United States, IIH demonstrates several sociodemographic disparities, with higher incidence rates observed among low-income individuals, Black populations, those aged 18 to 44 years, and females [18]. These findings underscore the intricate interplay of socioeconomic and geographic factors in shaping the prevalence of IIH across different regions. Further research is warranted to fully elucidate the causal links between race, ethnicity, access to healthy foods, exposure to unhealthy foods, and the occurrence of IIH. Understanding these complex relationships is crucial for developing targeted interventions to mitigate the burden of IIH and promote equitable healthcare outcomes across diverse populations.

Complications and prognosis

Neurological Sequelae Associated With IIH

Neurological sequelae associated with IIH encompass various symptoms and manifestations. Patients diagnosed with IIH often report experiencing severe daily pulsatile headaches, transient visual obscurations, pulse-synchronous tinnitus, photopsia, and retrobulbar pain. Less common symptoms may include diplopia, visual loss, and cranial nerve involvement, such as sixth or seventh nerve palsy. Papilledema, characterized by optic disc swelling resulting from increased ICP, is a hallmark sign of IIH and can lead to visual impairment if left untreated. Diagnosis of IIH typically involves a comprehensive approach, including a thorough patient history, detailed physical examination, and diagnostic imaging tests such as CT or MRI scans, along with a lumbar puncture to measure CSF pressure [5,22,23].

The treatment of IIH primarily focuses on weight management as a primary strategy. Even modest weight loss of approximately 5-10% can yield significant symptom relief. Medications such as acetazolamide, topiramate, and furosemide may be prescribed to reduce ICP by decreasing CSF production. In cases where conservative treatments prove ineffective, surgical interventions such as shunt surgery or optic nerve sheath fenestration (ONSF) may be considered. Complications associated with IIH include the potential for permanent visual impairment due to papilledema. While IIH is not typically life threatening, it can significantly impact individuals’ QOL as a chronic condition [5,8].

Long-Term Outcomes and Prognosis Factors

Predicting long-term outcomes in IIH poses challenges due to the condition’s complex nature. Various factors influence the prognosis of IIH patients, including sex, ethnicity, degree of papilledema, presence of transient visual obscurations, baseline visual acuity, and headache severity associated with IIH. Notably, weight loss has emerged as a critical modifier of IIH progression, as evidenced by the IIH Weight Trial demonstrating the benefits of bariatric maintenance for patients [24,25]. Studies have reported IIH relapse rates ranging from 9% to 28% in affected individuals. Pharmacological interventions like acetazolamide and topiramate are commonly employed to manage symptoms, while surgical interventions such as ONSF or shunt surgery may be warranted in severe cases. The recurrence of symptoms following recovery underscores the importance of regular follow-up visits for monitoring and management [24,26].

Despite the challenges, the prognosis for visual outcomes in IIH is generally favorable, with severe visual loss occurring in a small percentage of cases. Visual field abnormalities, including enlarged blind spots and generalized constriction, are common in IIH patients and may indicate significant nerve fiber layer loss. Timely diagnosis and appropriate treatment prevent irreversible visual damage in IIH patients [24,26]. Understanding the longitudinal course of IIH is crucial for optimizing patient outcomes. Extensive prospective cohort studies are needed to evaluate the real-world clinical progression of IIH comprehensively. Factors such as weight gain, severity of papilledema, and headache burden at disease onset serve as key prognostic indicators that can inform treatment strategies and enhance the management of IIH patients [25,26].

Impact on QOL and Functional Impairment

IIH affects affected individuals’ QOL. Studies have consistently demonstrated that patients with IIH experience diminished QOL compared to the general population, particularly in domains related to physical health. Vision-specific and overall QOL are notably lower in IIH patients at baseline, with significant disparities observed across various domains compared to the general population [27,28].

The severity of headaches emerges as a pivotal determinant of QOL among individuals with IIH. The resolution of headaches has been associated with tangible improvements in QOL, underscoring the imperative of addressing this debilitating symptom in treatment strategies. While improvements in clinical measures are undoubtedly significant, effective headache management is paramount to enhancing the QOL of IIH patients. This underscores the necessity of adopting a comprehensive approach encompassing specific headache treatments alongside traditional interventions targeting visual parameters [27].

Additionally, research suggests that weight loss interventions can yield notable enhancements in QOL for individuals grappling with IIH. Effective management of headaches and successful weight loss endeavors correlate with improved QOL outcomes, highlighting the multifaceted nature of treatment strategies for this complex neurological disorder. Despite strides made in understanding IIH and its impact on QOL, further research is warranted to delve into additional factors influencing QOL and functional impairment among patients contending with this challenging condition [25].

Management approaches

Medical Interventions

Pharmacological therapies: Acetazolamide is the foremost medication used to treat IIH. As a carbonic anhydrase inhibitor, it effectively reduces CSF production and lowers ICP. Research has demonstrated that a six-month course of acetazolamide, combined with a low-sodium weight reduction diet, yields modest reductions in ICP, improvements in QOL, and reductions in papilledema [29]. Topiramate is another medication utilized for IIH management. It is an anticonvulsant that decreases ICP and enhances visual function in select patients [30]. Furosemide, a diuretic, also serves as a treatment option by diminishing CSF production and lowering ICP [30]. While medications like amiloride and bumetanide have been employed in some instances to manage IIH, their efficacy still needs to be established [30]. The selection of medication hinges on factors such as the patient’s response to initial treatments, the comorbidities, and the severity of symptoms. A multidisciplinary approach involving ophthalmologists, neurologists, and neurosurgeons is strongly advised to optimize outcomes for individuals with IIH [30].

Lifestyle modifications: Weight management is paramount in IIH management, given obesity’s significant association with the condition. Patients are encouraged to maintain a healthy weight or embark on weight loss journeys when necessary [31]. Adopting a low-sodium diet is particularly recommended for obese IIH patients, as it may contribute to reductions in ICP [31]. Regular exercise regimens are strongly advocated alongside weight reduction diets [31]. Patients should also discontinue any exogenous agents that may elevate ICP [31]. While acetazolamide remains a cornerstone treatment for reducing CSF production and ICP, other medications, such as topiramate and furosemide, may also be considered [32]. In certain instances, individuals with mild symptoms or inadequate responses to initial treatments may be managed conservatively with regular monitoring and follow-up appointments [33]. Treatment selection is guided by symptom severity, the presence of comorbidities, and the patient’s response to initial interventions. Collaboration among ophthalmologists, neurologists, and neurosurgeons is essential to optimize outcomes for patients grappling with IIH [34].

Surgical Options

Shunting procedures: The lumboperitoneal shunt is a first-line surgical intervention for patients diagnosed with IIH. Despite the absence of prospective randomized studies, it is frequently utilized to divert CSF and alleviate ICP [35]. This standardized protocol for shunting procedures in IIH patients aims to reduce the need for revisions within 30 days of primary shunt surgery, thereby enhancing outcomes and minimizing associated complications [36]. Shunting involves the insertion of a hollow tube into the brain or spine to drain excess CSF, thus regulating ICP and alleviating IIH symptoms [37]. While recommended for only a minority of IIH patients, shunt surgery can significantly improve QOL by relieving symptoms [38]. Neurosurgical CSF diversion procedures manage elevated ICP in IIH, offering a range of interventions to redirect CSF flow and mitigate associated symptoms [39]. Individual patient characteristics, symptom severity, and response to non-surgical treatments guide the selection of a specific surgical procedure.

ONSF: ONSF is a surgical procedure designed to alleviate optic nerve swelling, particularly in cases of rapid or progressive vision loss associated with IIH. During ONSF, a small opening is created in the optic nerve sheath to allow excess CSF to drain, thereby reducing pressure and swelling. The surgery, typically performed under general anesthesia in an operating room setting, aims to safeguard eyesight from further deterioration, with most patients experiencing improved vision post-surgery [40,41]. Preoperative assessments and discussions with the surgical team are conducted to inform patients of the risks and benefits associated with ONSF. This procedure can be performed on one or both eyes, depending on the patient’s condition. Alternative surgeries, such as shunts, may be considered when ONSF is deemed unsuitable or ineffective [42,43].

Bariatric surgery in IIH with obesity: Bariatric surgery has emerged as a promising treatment option for patients with IIH and obesity. Studies suggest that bariatric surgery can lead to significant improvements in IIH symptoms, including headaches, visual disturbances, and papilledema. While the available evidence is limited in quality, there is support for the effectiveness of bariatric surgery in alleviating IIH symptoms and reducing ICP. Some studies have reported overall symptom improvement and disease remission following bariatric surgery-induced weight loss, with reductions in body weight ranging from 3% to 15% associated with remission [44-47]. Further research, including randomized controlled trials and cost-benefit analyses, is warranted to ascertain the efficacy, safety, and feasibility of bariatric surgery in severely obese patients with IIH.

Novel and Emerging Treatments

Recent advancements in treating IIH have ushered in novel approaches to managing this challenging condition effectively. Among these innovations, venous sinus stenting has emerged as a promising therapeutic option for patients with IIH. This procedure has demonstrated notable efficacy in improving affected individuals’ papilledema, visual fields, and headaches. Studies have consistently reported favorable outcomes with venous sinus stenting, including high rates of headache resolution, visual improvement, and low incidences of treatment failure and complications. As such, venous sinus stenting is increasingly recognized as a first-line surgical intervention for cases of IIH that are refractory to medical management [48-50]. In addition to venous sinus stenting, other emerging treatments for IIH include CSF diversion techniques, ONSF, and bariatric surgery for weight loss. These interventions share the common goal of reducing ICP, alleviating headaches, and preserving vision in patients with IIH. However, venous sinus stenting is a particularly significant advancement in the surgical management of IIH due to its efficacy and favorable safety profile [48,51,52]. As research in this field continues to evolve, these innovative treatment modalities promise to improve outcomes and enhance the QOL for individuals living with IIH.

Challenges and controversies

Diagnostic Challenges and Controversies

Navigating the diagnostic challenges and controversies surrounding IIH requires addressing various aspects of its intricate presentation and management. The diagnosis of IIH relies on identifying papilledema, excluding secondary causes, and confirming elevated CSF pressure. However, detecting papilledema can pose challenges, particularly in obese individuals. Differential diagnosis is pivotal in ruling out secondary causes of increased ICP, such as venous sinus thrombosis, anemia, obstructive sleep apnea, and medication-related factors like tetracyclines or excessive vitamin A intake. Moreover, the interpretation of abnormal lumbar puncture opening pressure remains controversial among clinicians [51,53,54].

Recent advancements in understanding IIH have shed light on the expanding clinical spectrum of the condition. Atypical presentations of IIH, including highly asymmetric or unilateral papilledema, IIH without papilledema, and cranial nerve involvement, are increasingly recognized. Radiological signs indicative of intracranial hypertension are also being identified in IIH patients, offering further insights into the underlying pathophysiology. Familiarity with these diverse presentations is crucial for clinicians to ensure accurate diagnosis and implement appropriate management strategies [8,55].

Treatment Dilemmas and Uncertainties

Treatment dilemmas and uncertainties in IIH stem from several factors, including a lack of understanding of the underlying pathogenesis, the limited availability of disease-modifying therapies, and the necessity for evidence-based management strategies. Consensus guidelines on IIH management underscore the importance of addressing the root cause of the disease, safeguarding vision, and minimizing the impact of headaches [56]. However, ongoing debates persist regarding the interpretation of abnormal lumbar puncture opening pressure, the influence of obesity stigma on treatment decisions, and the selection of optimal surgical interventions for severe IIH cases [30,56]. Additionally, the escalating economic burden associated with IIH underscores the urgent need for clear guidance to enhance patient outcomes and alleviate healthcare costs [56]. Addressing these treatment dilemmas and uncertainties requires a collaborative effort among clinicians, researchers, and policymakers to develop comprehensive and effective strategies for managing IIH.

Research Gaps and Future Directions

Research on IIH has identified crucial gaps and outlined future directions for investigation. Several key research priorities for IIH have been identified through collaborative efforts involving patients and healthcare professionals. These priorities encompass understanding the etiology of IIH, elucidating the mechanisms underlying headaches in IIH, exploring novel treatment modalities, investigating differences between acute and gradual visual loss, devising methods for monitoring visual function, identifying biomarkers of the disease, examining hormonal influences, exploring drug therapies for headaches, and assessing the role of weight loss, among others [57]. Furthermore, preclinical research focusing on ICP regulation in the context of IIH has underscored the need to explore novel drug targets for IIH treatment and deepen our understanding of the pathophysiology of elevated ICP in this condition. Studies have revealed that conventional drugs, such as acetazolamide, may offer limited efficacy in reducing ICP in IIH patients. This underscores the necessity for ongoing preclinical investigations utilizing refined methodologies to advance our comprehension of IIH pathophysiology and expand treatment options [58].

## Conclusions

IIH presents a complex clinical challenge characterized by increased ICP without an identifiable cause. This condition predominantly affects young obese women but can manifest in individuals across various demographics. Recognizing the symptoms of IIH, including headaches and visual disturbances, is crucial for timely diagnosis and intervention to prevent vision loss and other severe complications. Current management strategies involve a combination of medical and surgical interventions to reduce ICP and alleviate symptoms. Moving forward, efforts should be directed toward standardizing diagnostic criteria, increasing awareness among healthcare professionals, and fostering multidisciplinary collaboration to optimize patient care. Additionally, further research is needed to better understand the underlying mechanisms of IIH and develop more effective treatment approaches to improve outcomes and QOL for affected individuals.

## Figures and Tables

**Figure 3:**
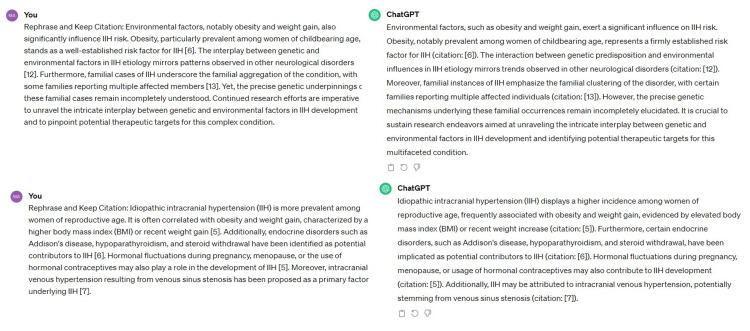
ChatGPT-assisted editing and rephrasing of the review article

## Appendices

The ChatGPT language model (OpenAI, San Francisco, California, United States) was employed to assist in formulating key arguments, structuring the content, and refining the language of our manuscript. It provided valuable insights and suggestions throughout the writing process, enhancing the overall coherence and clarity of the article. It was also utilized to assist in editing and rephrasing the work to ensure coherence and clarity in conveying the findings (Figure 3).
